# Travelling Editorials and Travelling Editors

**Published:** 1843-09

**Authors:** 


### TRAVELLING EDITORIALS AND TRAVELLING EDITORS.

We perceive that our contemporary of the *Boston Medical and Surgical Journal*, has been writing travelling editorials. We had the pleasure of meeting with him a short time since, among what a familiar of ours calls the "madcap levellers of the west;" and, faith, we were taken all a-back (we *must* perpetrate a sea phrase now and then) by seeing such a *veteran* in medical journalism looking *so* young. Not, indeed, that we had any sort of idea that he was "sightless, and old, and melancholy;" but we certainly *did* expect to see the crow's feet about the eyes, and mayhap something of a "frosty pow." We comfort ourselves for such a disappointment by reflecting that he will come up to our expectations by and by, and that meanwhile we shall see him here again next summer. May he live a thousand years, and may his shadow never be less! C.

